# Modelling and Analysis of the Corrosion Characteristics of Ferritic-Martensitic Steels in Supercritical Water

**DOI:** 10.3390/ma12030409

**Published:** 2019-01-28

**Authors:** Yanhui Li, Tongtong Xu, Shuzhong Wang, Balazs Fekete, Jie Yang, Jianqiao Yang, Jie Qiu, Aoni Xu, Jiaming Wang, Yi Xu, Digby D. Macdonald

**Affiliations:** 1 Key Laboratory of Thermo-Fluid Science and Engineering of MOE, School of Energy and Power Engineering, Xi’an Jiaotong University, Xi’an 710049, China; yhli19@sina.com (Y.L.); ttxu17@sina.com (T.X.); yangjianqiao@stu.xjtu.edu.cn (J.Y.); 2 Departments of Nuclear Engineering & Materials Science and Engineering, University of California at Berkeley, Berkeley, CA 94720, USA; balazs.fekete@berkeley.edu (B.F.); mengchunyj@gmail.com (J.Y.); qiujie@berkeley.edu (J.Q.); aonixu@berkeley.edu (A.X.); wangjiaming@fudan.edu.cn (J.W.); xuyi@berkeley.edu (Y.X.)

**Keywords:** F-M steels, artificial neural network, fuzzy curve analysis, supercritical water, oxidation, weight gain

## Abstract

The dependencies of weight gain of 9-12 Cr ferritic-martensitic steels in supercritical water on each of seven principal independent variables (temperature, oxygen concentration, flow rate, exposure time, and key chemical composition and surface condition of steels) have been predicted using a supervised artificial neural network (ANN). The relative significance of each independent variable was uncovered by fuzzy curve analysis, which ranks temperature and exposure time as the most important. The optimized ANN, not only satisfactorily represents the experimentally-known non-linear relationships between the corrosion characteristics of F-M steels and the key independent variables (demonstrating the effectiveness of this technique), but also predicts and reveals that the effects of oxygen concentration on the weight gains, to a certain degree, is influenced by the flow rate and temperature. Finally, according to the ANN predicted-results, departure of oxidation kinetics from the parabolic law, and basic cause of chromium content in steel substrate influencing the corrosion rate, and the synergetic effects of dissolved oxygen concentration, flow rate, and temperature, are discussed and analyzed.

## 1. Introduction

Energy, especially electricity, plays a vital role in our modern industrial society and in our current civilization. In order to increase the power generation efficiency of fossil fuels, supercritical and ultra-supercritical power plants, using supercritical water (SCW, temperature > 374.15 °C and pressure more than 22.1 MPa) as the working medium, have been developed over the past century [1]. Additionally, in the early part of this current, 21st century, the supercritical, water-cooled (nuclear) reactor (SCWR) has been envisioned as one of the six Generation IV nuclear power plant designs, because of its simplified core and higher thermal efficiency (45% vs. 33% in current light water reactor, LWR) [2]. However, compared to the water/steam environments that are commonly used in traditional nuclear reactors (LWR, PWR_pressurized water reactor), SCW is expected to be more corrosive, therefore the corrosion resistance of candidate structural materials becomes a key requirement. Because of relatively excellent properties, Ferritic-Martensitic (F-M) steels are primary candidates for the SCWRs design and are also currently used as common structural steels in fossil fueled thermal power plants [3,4]. The available literature reported a series of studies on the corrosion characteristics of F–M steels in SCW, from the oxidation kinetics [4,5,6,7,8], environmental parameters effects on corrosion [4,5,6,8,9,10], oxide scale formation and properties [8,11],and oxidation mechanisms [8,9,10,12]. For common F-M steels (~9–12 wt.% of Cr content) in SCW at 400–700 °C, the oxidation process generally follows near-cubic or near-parabolic rate laws [4,13]. They are unambiguous whether high temperatures can accelerate the oxidation process [4,6,8], and that high chromium content within the substrate and oxide dispersion strengthening treatment are known to be beneficial for improving the oxidation resistance of the F-M steels [7,9,10]. However, no consensus has been reached concerning the effects of oxygen concentration and flow rate of SCW on the oxidation behavior. Zhang et al. indicated that the weight gain increased with increasing dissolved oxygen content from 100 to 300 to 2000 ppb [14]. However, the results are inconsistent with those of Ampornrat et al. [4] who concluded that oxygen concentrations of 150–200 ppb resulted in lower weight gains than either lower or higher oxygen contents. These inconsistences may be because of differences in the experimental conditions. The effects of oxygen concentration on the oxidation kinetics of F-M steels, to a great degree, may be greatly influenced by the experimental temperature and flow rate of the corrosive medium. Furthermore, the effects of oxygen concentration, flow rate, and the physic-chemical properties of SCW, all belonging to the relevant environmental factors, have been seldom varied systematically in previous studies and need to be explored to deepen our understanding of the intrinsic corrosion mechanisms of the alloys.

In addition, in order to maximize the corrosion resistance of service materials, in view of whether optimization and design of the potential F-M steels for constructing SCWRs or by control various water chemical parameters to mitigate the material degradation, a near quantitative relationship between the alloys’ corrosion characteristics and various parameters, such as the key chemical composition and structure of alloys and the service conditions (temperature, oxygen level, etc.), is necessary for engineering and materials selection and design purposes. A high quality, a representative model would provide a favorable method for environmental parameter control, help to develop an optimum design strategy for the required alloys, and spare researchers from having to carry out infinite measurements to resolve technical issues. However, a rigorous mathematical treatment of the problem is almost impossible because of the inherent complexity of the processes involved. In other words, we see no way of using the available theories of materials, corrosion, and metallurgy and their attendant deterministic models to develop a comprehensive, quantitative description of the corrosion kinetics of an alloy given a chemical composition and structure and the various environmental parameters. Therefore, it is necessary to develop a new path toward this goal.

The most appropriate, logical selection is to ascertain the dependent variable (weight gains)/independent variable relationships by artificial neural network (ANN) modelling [15]. An ANN is basically an intelligent data-driven black-box model, not requiring a mathematical description of the phenomena involved in the process, and is capable of capturing the highly complex, non-linear input/output relationships [16,17]. A key advantage of the ANN is fault-tolerance and the ability to learn, recognize, generalize, and interpret the impact of incomplete and noisy inputs. In the training phase, the knowledge about a given process contained in known data will be acquired and then stored in numerical form within the ANN model [17] via optimization of the weights of the connections between neurons. The trained ANN can not only identify hidden relationships between the dependent and independent variables (‘tells it the way it is’), but can also forecast the previously unknown dependencies of outputs on inputs from the learned relationships (‘predict what will most likely happen’). In the field of corrosion prediction, successful neural network approaches have been employed and reported by a number of researchers [15,16,17,18]. ANN modelling had helped our research team to reveal that the intergranular stress corrosion cracking in sensitized type 304SS and alloy 600 in a high-temperature aqueous environment is primarily electrochemical and not mechanical in character [15,17]. However, applications of this technology in corrosion studies of alloys in supercritical water, especially for 9-12Cr F-M steels, are rare or non-existent.

Using ANN modelling and fuzzy curve analysis, this paper aims to explore the effects of various independent variables (temperature, chromium content, oxygen concentration, flow rate, composition and surface conditions of steels) on the weight gain of 9-12Cr F-M steels in supercritical water, and to categorize the importance of each independent variable to the output. The logical structure of this paper is as follows. Firstly, Section 2 introduces the basics of artificial neural network, deals with the whole process of developing ANN model for the weight gain of F-M steels in SCW with seven independent inputs, from initial data collection to finial verification of obtained model, aiming to model the corrosion characteristics; and then clarifies the basic algorithm of fuzzy curve analysis being employed to identify the significant inputs. Section 3.1 gives that fuzzy curve analyses ranked temperature, exposure time, and Cr content within the steel substrate as the most important inputs. Therefore, in Section 3, except Section 3.1, depending on the optimized ANN model, this paper predicts and explores the effects of the above three key input parameters and the dissolved oxygen content on the weight gains of F-M steels in supercritical water. On this basis, special emphasis are given to discussion and analyses on the departure of oxidation kinetics from the classical parabolic law, the role and method of chromium in hindering the outward transport of metal cations, and the synergetic effects of dissolved oxygen concentration, flow rate, and temperature.

## 2. ANN and ‘Fuzzy Logic’ Analyses

### 2.1. Neural Network Backpropagation Method

**Figure 1:**
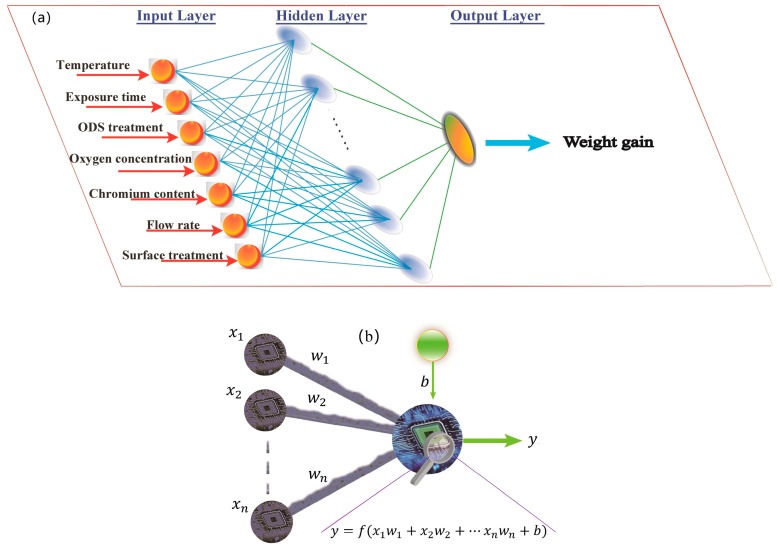
(**a**) A three-layer network used for prediction on the weight gain and (**b**) the processing information schematic of a single artificial neuron.

Inspired by the operations and connectivity of the biological neural networks, such as the brain, an ANN is a computational tool that is capable of capturing the highly complex, non-linear inputs/output relationships that exist in complex systems. The ANN comprises an array of highly interconnected, processing nodes, i.e., artificial neurons [16,17,19]. A general schematic diagram of a three-layer network (an input layer, a hidden layer, and an output layer in sequence) is illustrated in Figure 1a. Each layer comprises one or more neurons. An elementary neuron acts as a microprocessor with input and output sides, in which a transfer function and a bias exist, as shown in Figure 1b. Corresponding to the dendrites of biological neurons, the connections on the input side receive signals from the neurons within the preceding layer or the original database, while the output connections are analogous to the biological neuron axons. The outputs of some of the neurons in the preceding layer are the inputs to the following layer or are the dependent variables that we need. Each neuron of the input layer (first layer), all with only one input connection, directly receives signals from the user’s self-defined database and transmits them as the neuron’s outputs without any change. As for the output layer, i.e., the last layer, their output values correspond to the dependent quantities that the user seeks to know. A strength of the connection between two artificial neurons is defined as the *weight*, which is generally assigned a negative value (inhibition) or a positive value (excitation). The absolute values of the weights are generally between ‘0′ and ‘1′. A higher weight corresponds to a stronger connection. As mentioned previously, the neuron also contains the transfer function and the bias. The neuron sums the weighted inputs (products of each input and its corresponding *weight*) and the bias, and then the obtained result is applied to form the input to the single-value transfer function, which generates the neuron’s output.

Mathematically, assuming that one neuron with a bias of *b* and a transfer function, *f*, receive an n-dimensional input vector, *x* = (*x*_1_, …, *x_n_*), of which the *weights* scaling the respective input values to the neuron are *w* = (*w*_1_, …, *w_n_*), the output of the neuron (*y*) is expressed and shown in the above diagram (Figure 1b).

In fact, the *weights* and *bias* presented in the ANN just reflect the ‘knowledge’ stored in a biological neural network, such as the human brain. It is well-known that every brain has the amazing ability to adapt itself by forming and/or improving connections between brain cells (biological neurons) based on previous experience and knowledge, to render itself “smarter” (capable of performing more complex tasks more rapidly). Similarly, in order to develop a well-performing ANN, it is necessary to train the network, i.e., “let it learn”, by optimizing *weights* and *biases*. The learning algorithms of artificial neural networks can be divided into supervised and unsupervised methods [18,19]. Backpropagation, short for “backward propagation of errors,” is a general adaptive algorithm for supervised training of ANNs using the gradient descent optimization method. For this kind of training, a set of input/output data pairs, that is in the form of a randomly preassigned “training set” database culled from the entire database, is directly presented to the input layer, and passed on to the output layer. The outputs computed by the network are observed and compared with the target outputs, to obtain the difference (generally in mean square error-the average squared error, *mes*, as follows) between them. Depending to the calculated error, the backpropagation training algorithm iteratively adjusts the biases and weights of the various neurons from the output layer to the input layer until the predefined minimum error is obtained or some other termination criteria are reached. In order to avoid overfitting, a “validation set” dataset is employed to monitor the training process and identify the stop point. The third part of the original database, a ‘test set’, is also necessary and serves to determine the forecast performance of the trained network. The readers can obtain additional information on the ANN backpropagation training method from a reference book [19].

(1) $$mes=\frac{1}{N}\overset{N}{\underset{i=1}{\sum }}{\left(e_{i}\right)}^{2}=\frac{1}{N}\overset{N}{\underset{i=1}{\sum }}{\left(t_{i}-y_{i}\right)}^{2},$$

where *N* is the total number of the samples, and $t_{i}$ and $y_{i}$, respectively, are the outputs and targets.

### 2.2. Data Collection and Preprocessing

As the first step in performing ANN modelling, the collection of data, is critical to the success of the future trained model. The identification of key variables and generation of an available database is the primary and critical step for developing a properly functioning network. All of the noteworthy parameters that influence the weight gain of F-M steels in SCW (weight gains), generally can be divided into two groups: The composition and structure of the material, and experimental factors (temperature, oxygen concentration, pressure, flow rate of medium, etc.). Following on our previous experimental studies on F-M steel in SCW [8] and from the available literature [4,5,6,7,8,9,10,14], we identified that, in addition to temperature (*T*), chromium content in the substrate (*Cr*%) and oxygen concentration (*DO*, dissolved oxygen), flow rate of medium (*v*), surface treatment (initial or grain-refinement surface, *ST*), and the presence of dispersed oxide strengthening additions (*ODS*) also play a significant role. Thus, the above six fundamental variables and exposure time are included in our database, in order to simulate the corrosion characteristics of 9-12Cr F-M steels in SCW. For the first three parameters (*T*, *Cr*%, and *DO*), generally as the commonly-controlled parameters in experimental studies, they are usually available in the published literature. However, in order to unify the other three parameters from various sources and/or as implied in the reporting papers in different forms, and as required of the inputs for an ANN, we calculated and defined these variables according to the available information contained in the original papers or reports. In the case of flow rate, it was designated as being zero if the experimental work was performed in a static autoclave [14,20]. For a series of studies performed at the University of Wisconsin-Madison in Madison, WI, USA, a flow rate of ~1 m/s in their SCW-loop at 500 °C was reported [7], thus the flow rate at a variety of temperatures under similar mass flux of the inlet water can be estimated. As for surface condition (*SC*), we focused mainly on whether or not grain refinement occurred in the surface layer of the substrate. The *SC* is assigned to “1” if grain refinement treatment exists, otherwise *SC* = ‘0′. In available literature or published reports, the tested oxide dispersion strengthened (*ODS*) steels usually are produced by mechanically alloying with Y_2_O_3_ or Y_2_Ti_2_O_7_ dispersion particles. The content of oxide dispersion particles generally falls into the range of 0.3 wt.% to 0.4 wt.% [21,22,23]. Due to the oxide type and content were relatively stable and unitary within the collected database, so the authors did not distinguish the small difference, and employed simple binary method: The ODS input was designated to “1” if the oxide dispersion strengthening exists, otherwise it was “0”. Table 1 shows the involved input variables and their ranges within the available database.

Another important step before training and ‘refining the network’, is cleaning (pre-processing) the original data using the appropriate statistical tools that are available to avoid the dominance of one variable that has an exclusively larger value. The common method is that the initial data are normalized between 0 and 1. Herein, the database (*x*_i_) were linearly scaled to a new set (*X*_i_) by the equation below:

(2) $$X_{i}^{k}=\frac{x_{i}^{k}-min_{i=1,\ldots ,N}x_{i}^{k}}{max_{i=1,\ldots ,N}x_{i}^{k}-min_{i=1,\ldots ,N}x_{i}^{k}},$$

where *k* represents the seven independent variables (*k* = 1, 2, …, 7) and the targeted independent variable, and *N* stands for the amount of data in the data set.

### 2.3. Model Formulation

**Figure 2:**
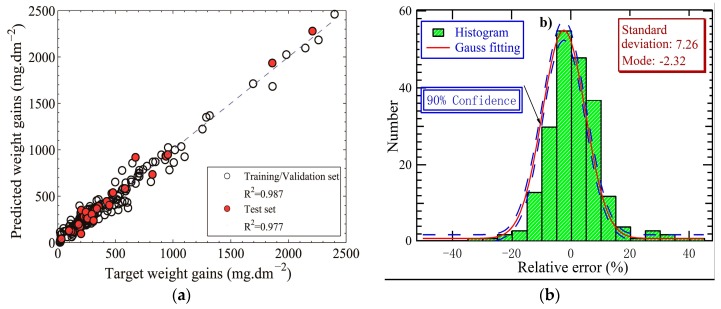
(**a**) Neural network predicted versus target outputs, and (**b**) statistics on the relative errors of artificial neural network (ANN) predictions.

The building and construction of an ANN is done to facilitate identification of the relationships between the outputs and every independent input variable, imbuing the net with ‘‘intelligence’’. In the present case, after screening, unification, and normalization, the database contained 254 experimental data that, simultaneously, contain the outputs (weight gain), as well as values for the independent variables, and that does not obviously deviate from the expected outputs depending on the abundant experimental results. Before training commenced, the dataset was randomly divided into three subsets: A training set (70%), a validation set (15%), and a test set (15%). A three-layer, feed-forward ANNs with one hidden layer were chosen, as depicted schematically in Figure 1a. It has been suggested that, by universal approximation theory, a network with one single hidden layer with a sufficiently large number of neurons can interpret any input–output mapping structure [24]. The number of neurons in the input and output layers are determined by the number of input parameters (seven) and the number of expected outputs (one). The selection of neurons in the hidden layer plays a significant role in network performance. However, there is no general and widely recognized formula for determining the optimum number of hidden neurons but the number that is chosen generally is that which gives the best performance (Minimum error in the output with respect to the quality of the input data). In this paper, a group of neural networks with hidden neurons of 10 to 36 with an interval of 2 were trained and evaluated on the test data set. In this approach, the ANN with the highest R^2^ (lowest error) and the lowest number of neurons in the hidden layer (implying enough generalization capability) should be a good choice. Although this approach may be time consuming, especially when dealing with a great abundance of samples, it generally works very well. In this paper, the ANN was trained using the Levenberg–Marquardt backpropagation (LMBP) algorithm. The logistic sigmoid transfer function was employed in each neuron in the hidden layer. A linear output layer, which is most often used for function fitting problems, was employed. All of the calculations were carried out using the Matlab2013b Neural Network Toolbox developed by MathWorks (Natick, MA, USA). According to the experimentation results, a 30-neuron hidden layer (i.e., a 7:30:1 architecture) was chosen. Plotting predicted outputs by target outputs, Figure 2a illustrates the R-squared value, in which the training/validation datasets were fitted relatively well with R^2^ = 0.987 and the test set was predicted with good performance (R^2^ > 0.97). In addition, Figure 2b gives the statistic distribution of the relative errors which are defined as the ratio of differences between ANN-predicted and target outputs to the target outputs, and the satisfying gauss fitting results. For more than 90% samples of the whole dataset, the relative errors of ANN predictions were within ±12%, further identifying the reasonable accuracy of the obtained ANN model, which also does uncover non-linear relationships between dependent and independent variables, as provided in Section 3.

### 2.4. Fuzzy Curve Analysis

As a purely mathematically empirical model, artificial neural network are often criticized for their “black box” property, in that they are unable to reveal a mechanistic governing the physical processes and laws that govern the dependencies of the outputs on one or more of the inputs [18]. Therefore, it may be of great importance to obtain insight into the various degrees of significance of the various inputs on the outputs, to help exhibit the connotative logic relationships between the inputs and outputs within the trained ANN (i.e., to develop “knowledge”). Several methods, such as sensitivity analysis, fuzzy curve analysis, and change of mean square error, are available to determine the impact of a range of independent variables on one particular dependent variable as established using a given dataset [15,17,18,25]. Fuzzy curve analysis derives from fuzzy logic, which represents a type of many-valued logic that aims at handling the concept of partial truth (generally, the truth value may range between completely true and completely false), and is closer to human logical intuition [25]. Sung comparatively analyzed the effectiveness of the above mentioned methods in ranking the importance of input parameters, indicating that, in most case, the fuzzy curve method performs better than sensitivity analysis and the change of mean squared error approach [26]. Additionally, the fuzzy curve analysis has been employed widely and has proven to be effective in identifying the contributions of the independent variables to the dependent variable [17,18].

Based on the fuzzy logic algorithm, fuzzy curve analysis, which was first proposed by Lin and Cunningham [25], was employed to identify the significant inputs and determine the proper model structure for a multiple-input, single-output system. Assume that a system has *N* independent inputs and *M* sample points are available, *x_i,j_* (*I* = 1, 2, …, *N*; *j* = 1, 2, …, *M*) represents the *i*th input of *j*th training sample and the corresponding output is *y_j_*. For the implementation of fuzzy curve analysis, it is first necessary to calculate the fuzzy membership vector (total length of *M*), $\varphi_{i,j}$, for each input variable *x_i_*. Any fuzzy membership function, such as Gaussian, Triangle, Trapezoidal, and Gbell, can be employed to map the input to the elements of the set [0,1], although Gaussian is more popular, as follows.

(3) $$\varphi_{i,j}=\exp\left(-\frac{{\left(x_{i,j}-x_{i}\right)}^{2}}{b}\right),$$

where b is taken as approximate 20% of the length of the input interval of *x_i_*. The ordered pair of *y_j_*-$\varphi_{i,j}$ defines *M* fuzzy rules for *y* with respect to *x_i_* [25]. $\varphi_{i,j}$ can also be regarded as the degree of accuracy of *y* = *y_j_*, which is definitely true if $\varphi_{i,j}$ = 1, while it is unquestionably false if $\varphi_{i,j}$ = 0. The expected fuzzy curve *C_i_* for each input variable *x_i_* can be obtained by a defuzzification process with the *y_j_*-$\varphi_{i,j}$ pairs as the inputs, via the centroid calculation,

(4) $$C_{i}=\frac{\sum_{j=1,2\ldots N}\varphi_{i,j}\cdot y_{j}}{\sum_{j=1,2\ldots N}\varphi_{i,j}},$$

which returns the centroid of the $\varphi_{i,j}\;vs\;y_{j}$ plot. Thus, the fuzzy curves for each input *x_i_* can be separately plotted in the *C_i_*-*x_i_* space. They clearly depict the range spans of the output data on the *C_i_*-axis versus each input variable. The larger the range span are, the higher is the significance of the corresponding input variable [17,25,26].

## 3. Results and Discussion

### 3.1. Significance of Variables

**Figure 3:**
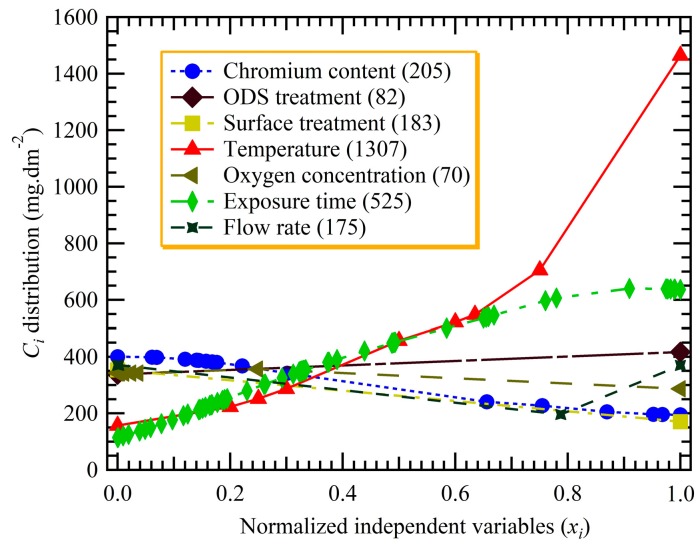
Fuzzy curves for the weight gain and its spanned ranges corresponding to each independent variable.

As noted above, according to the range span covered by the fuzzy curve of each input parameter, the relative importance of the independent (input) variables can be ranked with respect to their contribution to the dependent (output) variable. Here, for the convenience of plotting fuzzy curves of seven inputs on one graph, all of the input parameters were linearly normalized between 0 and 1, as shown in Figure 3. Figure 3 also indicates the range spans of each fuzzy curve in the legend. As seen in the figure, the importance of seven independent variables for the weight gain are ranked as follows: *T* > *t* > *Cr*% > *ST* > *v* > *ODS* > *DO*. Obviously, temperature and exposure time are of greatest significance. However, there are only slight variations in the significance among the other parameters except the ODS treatment and oxygen concentration. The oxygen concentration has the lowest significance, but is only slightly lower than the ODS treatment. In fact, it has been widely acknowledged that temperature generally has the greatest effect on the corrosion behavior of heat-resistance alloys [4,7,27,28,29,30,31]. Therefore, in order to evaluate an alloy’s performance in industry, it is generally the first step to consider the temperature effect. The considerable effects of temperature, exposure time, and chromium content are further explored below. It is noteworthy that the importance of oxygen concentration (10–8000 ppb) is in the last place. This result can be accounted for by the fact that the primary reactant is not O_2_ but is H_2_O, a finding that is in concert with that of Guan and Macdonald for the corrosion of austenitic stainless steels in SCW [32].

### 3.2. Temperature and Exposure Time

**Figure 4:**
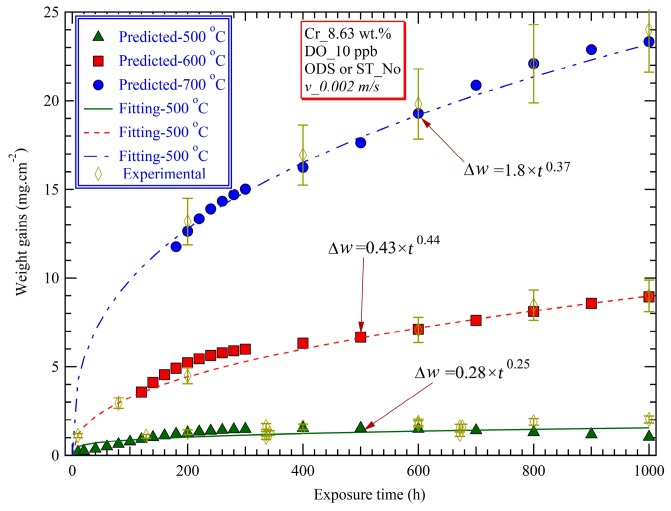
ANN-Predicted and experimental weight gains versus exposure time and the fitting curves.

Temperature is ranked as the most significant independent variables influencing the corrosion behavior of ferritic-martensitic steels [4,7,27,28,29] in SCW, as shown in Figure 3. The predicted weight gains as functions of exposure time at three typical temperatures of 500 °C, 600 °C, and 700 °C and fitted oxidation kinetics are plotted in Figure 4. The other necessary input parameters for the ANN prediction are given in the top center box in Figure 4. As the exposure time increases, the weight gain increases significantly in the initial stage, and then the increasing rate (dΔw/dt) decreases gradually and tends to a nearly constant value at sufficiently long times. Exposure at higher temperature leads to a more rapid increase in the weight gain. The weight gains at 700 °C are 2–3 times those at 600 °C, which are 4–6 times those at 500 °C [4,8]. The experimental data from References [13,20,33] are also shown in Figure 4, and excellent agreement exists between the ANN-predicted results and experimental data at 500 °C and 600 °C, but at 700 °C only for exposure times above approximately 200 h. The poor prediction ability of the ANN for the weight gains of F-M steels exposed for less than 200 h at 700 °C, not shown here, can be attributed to the few available experimental data included in the database for training.

The time dependence of the weight gains was assumed to follow an exponential law, $\Delta w=\sqrt{k_{p}}\times t^{n}$ ($k_{p}$ being rate constant at a given temperature), with time exponents of *n* = 0.25, 0.44, and 0.37 at 500 °C, 600°C, and 700 °C, respectively. The time exponents increase initially from 500 °C to 600 °C and then decrease to 0.37 when the temperature is raised to 700 °C. The oxidation kinetics follows a near-cubic law (with a time exponent of ~0.33) at the lower and upper extreme temperatures of 500 °C and 700 °C, while it follows near-parabolic oxidation kinetics at 600 °C. There is considerable experimental and theoretical evidences that the rate-controlling process in the total weight gains of the F-M steels exposed to SCW is the outward diffusion of iron cations [7,10,12,33], despite of the fact that the inner layer grows into the metal, which is only possible if oxygen vacancies are produced at the metal/barrier layer interface [34]. Even if the inner layer does grow via the generation of oxygen vacancies at the inner layer/metal interface, thereby resulting in an outward flux of oxygen vacancies and an inward flux of oxide ions, the outward flow of cations through the inner layer is responsible for the growth of the much thicker outer layer. According to the Wagner’s theory for thick film following the parabolic kinetics [35,36], the weight gain of alloys Δw with exposure time *t* becomes

(5) $$\Delta w={\left(k_{p}\cdot t\right)}^{1/2},$$

(6) $$k_{p}=\Omega^{2}\;\int_{a{\left(O_{2}\right)}_{I}}^{a{\left(O_{2}\right)}_{II}}\left[\alpha D_{M,eff}+D_{O,eff}\right]d\left[\ln\left(a_{O2}\right)\right],$$

where, $k_{p}$ should be the temperature-dependent oxidation rate constant, $D_{M,eff}$ and $D_{O,eff}$ stand for the effective diffusivity of the metal cations and the oxide ions (or oxygen vacancies) within the film ($MO_{\alpha }$), and Ω as the oxygen content of the formed oxides in a unit of mg·cm^−3^. The limits of integration in Equation (6) are the oxygen activities at the metal/scale interface [$a{\left(O_{2}\right)}_{I}$] and the scale/environment interface [$a{\left(O_{2}\right)}_{II}$]. Although these cation-diffusion models have been much-criticized [37,38] because of their failure to account for fundamental quantities (e.g., the “jump distance”) and their neglecting on the existence of space-charge regions near the metal/scale and scale/environment interfaces and because they fail to account for the behavior at limiting small times (finite rate) and limitingly long times (steady-state), they are still used somewhat uncritically since they “seem to work” and they, at the phenomenological level at least, can relate the growth rate of oxide film to other measurable transport properties of the oxide, such as diffusion coefficients. An alternative to these models is the Point Defect Model (PDM) [34], in which the growth of the inner layer is attributed to the inward transport, primarily by migration of oxide ions (outward transport of oxygen vacancies) and the outward transport of metal cations either via a cation vacancy mechanism or as interstitials through the inner (barrier) layer leading to the growth of the porous, generally water-transmissible outer layer. The PDM, which was developed at the atomistic level to describe the growth of multilayer, anodic oxide films in electrolyte solutions at subcritical temperatures, is now being extended to describe the growth of scales at supercritical temperatures [39].

Under a given corrosion system (i.e., constant [$a{\left(O_{2}\right)}_{II}$]), for the ideal parabolic kinetics, the parameter $k_{p}$ is definitely a constant, only being dependent on environmental temperatures. But in actual metal oxidation cases, due to these factors that A) formation of caves within the film, B) gradual loss of contact at the metal/oxide interface, C) presence and variation in density of short-circuit diffusion paths, such as dislocations and grain boundaries, etc., $k_{p}$ can be assumed to be related to the exposure time, as follows:

(7) $$k_{p}=k_{p,0}\times t^{2m},$$

where $k_{p,0}$ is a standard temperature-dependent constant, and *m* represents the dependence of $k_{p}$ on the exposure time. Comparing with the obtained kinetics, shown in Figure 4, it can be calculated that *m* equates −0.25 and −0.06 at 500 °C and 600 °C, respectively. The decrease in $k_{p}$ with the exposure time can be attributed to the decreasing $D_{M,eff}$ and $D_{O,eff}$ due to the above mentioned factors: A), B), C), and so on. The effective diffusion coefficient can be defined as the total diffusivity of volume diffusion and grain boundary effect [33,40]. On one hand, factors A) and B) go against the volume diffusion of cations and anions [36]. On the other hand, the grain boundary diffusivity decreases with an increase in oxide grain size when the exposure time extends [13]. These two sides, to a certain degree, provide reasonable explanations of the decrease of $k_{p}$ with exposure time. However, the diffusion of metal cations and oxide ions (oxygen vacancies) along oxide grain boundaries and the decreasing density of the short-circuit diffusion paths due to the enlarging of oxide grain size, plays a predominant role in the departure of oxidation kinetics from the parabolic law [33,36]. At higher temperatures, due to relatively high defect concentrations within the oxide scales, the significance of the grain boundary diffusivity weakens. Therefore, the higher the temperature is, the lower is the initial proportion of the grain boundary diffusion in the effective diffusivity. The dependence of the effective diffusivity on exposure time declines from 500 °C to 600°C. Gao et al. also reported that, at 600 °C and even higher temperature in SCW, the effect of grain boundaries on cation diffusion is not important, at most close to that of lattice transport [41,42]. However, when the temperature is raised to 700 °C from 600 °C, *m* decreases from −0.06 to −0.13, i.e., a higher departure from the parabolic kinetics, implying reoccurrence of predominant mode of the diffusion along “short-circuit” paths. On the one hand, this phenomenon may stem from local formation of protective chromia within scales due to enhanced selective oxidation of Cr at higher temperatures The chromium diffusivity in ferritic steel (a Cr content of 8.63 wt.%) is larger by approximately four orders of magnitude at 700 °C (~10^−18^ m^2^ s^−1^) than it is at 500 °C (~10^−22^ m^2^ s^−1^) [43]. Accordingly, it is likely that a considerable proportion of Cr_2_O_3_ is formed and present in the oxide scales at 700 °C, decreasing its defect density and lattice diffusivity. On the other hand, the occurrence of some micro cracks triggered by the growth stresses at the high temperatures [8,9,13], can enhance these short-circuit diffusion paths and therefore further weakens the volume diffusion advantage [1,23]. Finally, the possibility must be kept in mind that the parabolic law might not be applicable at all at extreme temperatures and that the discussion of grain boundary diffusion is nothing more than rationalization. Some attempts to overcome these disadvantages of the parabolic equation will be provided in a future article [39].

For the oxidation of F-M steels in SCW, depending upon the magnitude of the second derivative of Δ*w* with respect to exposure time (*d*^2^Δ*w*/*dt*^2^), the process generally can be divided into three stages: Rapid oxidation (predominantly nucleation of oxide particles, *d*^2^Δ*w*/*dt*^2^ > 10^−3^ mg cm^−2^ h^−2^), a transitory stage (formation and densifying stage of the protective Cr-rich inner layer, the formation of columnar oxide grains in the outer layer), and a relatively steady-state stage (*d*^2^Δ*w*/*dt*^2^ < 10^−5^ mg cm^−2^ h^−2^) [8]. According to the evaluated kinetic equations in the present study, the ending time of the rapid oxidation stage is ~10 h, ~15 h, and ~20 h at 500 °C, 600°C, and 700 °C, separately, which are located similar range to our previous experimental results that the rapid oxidation of T91 exposed to SCW at 540 °C finishes before ~20 h. Meanwhile the starting time of the steady-state stage at the three temperature defers to about 100 h, 400 h, and 420 h in turn. However, on the contrary, for austenitic steels serviced in SCW [28], when the exposure temperatures increased from 540 °C to 580 °C, the transitory and diffusion-controlled stages occurred earlier. These seemingly disparate results occurred on F-M steels and austenitic steels are essentially determined by the different formation mechanisms of the oxide scales during the early oxidation process [8,28]. For austenitic stainless steels in SCW, the continuous inner Cr-rich spinel layer was observed to form preferentially relative to the outer magnetite layer [28]. Higher temperatures can enhance outward diffusion of Cr within the substrate steel, accelerates the formation of a continuous, protective chromium-rich oxide layer, and results in the subsequent occurrence of the transitory- and steady state stages at shorter exposure times. While, during early oxidation, the complete outer layer comprising magnetite and the inner, Cr-containing layer formed almost simultaneously on F-M steels in SCW, i.e., the duplex structure scale (a ~1.5 um inner layer and an outer layer of ~1.5 um) was observed on T91 exposed to SCW at 540 °C for only 1 h [8]. An increase in temperature up to 600 °C, which is not only favorable to outward transport of Cr, but also enhances iron diffusion outward and inward transport of oxygen, accelerates the simultaneous thickening of the inner and outer layers and is not conducive to the formation of diffusion layer (general representing the arrival of steady state oxidation stage) [8].

### 3.3. Effect of Cr Content

**Figure 5:**
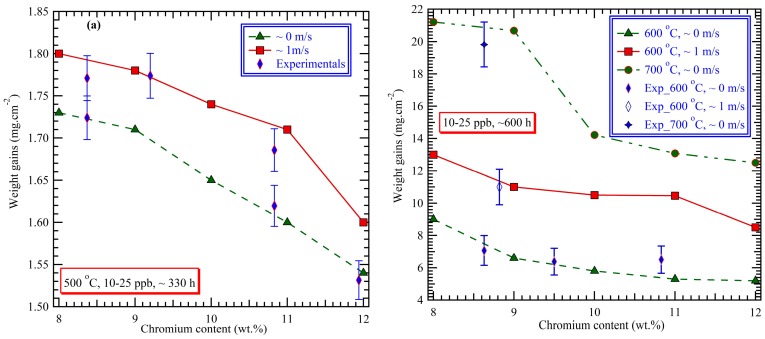
Predicted-weight gains of F-M steels vs. chromium content at (**a**) 500 °C, (**b**) 600 °C and 700 °C, as a function of flow rate.

Generally, the good corrosion resistance of the steels with higher Cr contents is expected compared to the steels of lower Cr contents. The weight gains of F-M steels were extracted from the trained neural network at 500 °C, 600 °C and 700 °C under the specific conditions by inputting various chromium contents of 8–12 wt.%. These weight gains are plotted as a function of the chromium content, shown in Figure 5 for various flow rate conditions. Overall, these predicted weight gains are in reasonable accord with the reported experimental results [10,13,44]. Regardless of the exposure temperature, the weight gain decreases with an increase in the chromium content. Relatively speaking, all of the steels with chromium contents of 8–12 wt.% have very similar oxidation resistance at 500 °C, with the difference in the weight gains, for the same exposure time of approximately 330 h, being less than 0.2 mg cm^−2^, as indicated in Figure 5a. However, the difference between the 9Cr steels and the 12Cr steels is only slightly apparent at 600 °C, but it becomes marked at 700 °C. Additionally, it is worth noting that when the oxygen content is 10–25 ppb, an increase in flow rate from approximate zero to slightly more than 1 m s^−1^ accelerates the oxidation of F-M steels, with the extent of acceleration being temperature-dependent.

**Figure 6:**
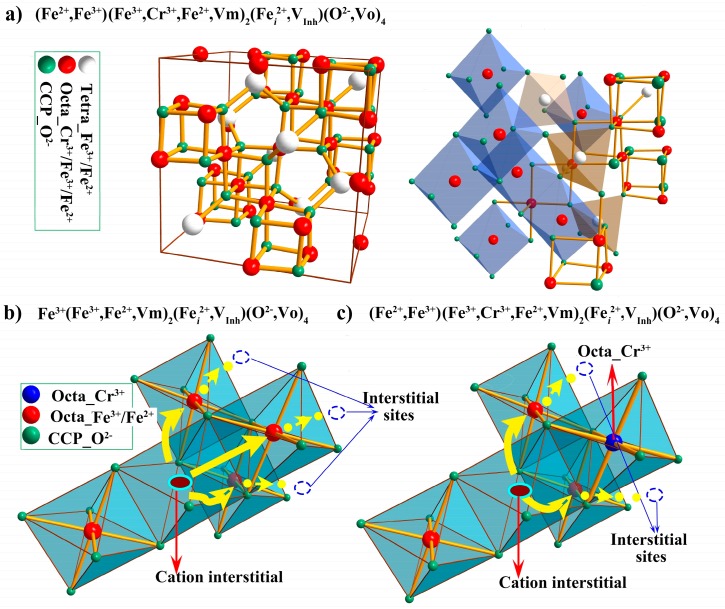
Schematics of (**a**) elemental distribution in a spinel Fe(Fe,Cr)_2_O_4_ cell and diffusion paths for cation interstitials within (**b**) magnetite and (**c**) Fe_3−x_Cr_x_O_4_ (some Cr^3+^ replacing Fe^3+^ in octahedral sites of magnetite). Note: In (Fe^3+^,Fe^2+^)(Fe^3+^,Cr^3+^, Fe^2+^,Vm)_2_(Fe*_i_*^2+^,V_Inh_)(O^2−^,Vo)_4_, the four items, in turn, represent tetrahedral interstices, octahedral interstices, extra octahedral interstices which are inherently vacant (V_Inh_) for the stoichiometric composition, and oxygen sublattices, respectively.

The inner and outer layers of oxide scales formed on F-M steels in SCW generally comprise Fe-Cr spinel (Fe_3−x_Cr_x_O_4_) and magnetite, respectively, which all can be described as face-centered cubic, close-packed sublattices of *n* oxygen ions with one eighth of the total of *2n* sites having tetrahedral coordination occupied by Fe^3+^ and Fe^2+^, and the half of the *n* octahedral coordination filled with cations (Fe^3+^, Fe^2+^,and/or Cr^3+^) [45,46]. At lower temperatures (less than 400 °C), magnetite has a unit cell of 32 O^2−^ ions into which are placed 8 Fe^3+^ ions at tetrahedral sites and 8 Fe^2+^ + 8 Fe^3+^ ions at octahedral interstices (i.e., inverse spinel structure), while a certain proportion of Fe^2+^ goes into the tetrahedral interstices, more like a normal spinel, with an increase in temperature [46]. Generally speaking, the principal defects are cation vacancies and iron interstitials, which is the basic supporting theory for the off-stoichiometric structure, as well as for the metal transport within this bulk oxide, while the oxygen sublattice remains relatively fixed because its defects have higher energy [11,46,47,48]. However, for oxide formation at the metal/oxide interface, and noting that the oxide grows into the metal, which is correct for the growth of the inner layer, then oxygen vacancies are always produced at the metal/inner layer interface leading to the inward transport of oxide ions, and hence are always present in the barrier layer regardless of their energy [49,50]. Without displaying any defects and without distinguishing between the different cations on the same coordination sites for simplicity, Figure 6a presents a schematic of the elemental distribution (left) and some octahedral/tetrahedral sites (right) in a complete unit cell of the forming (Fe^3+^, Fe^2+^)(Fe^3+^,Cr^3+^, Fe^2+^,Vm)_2_(Fe*_i_*^2+^,V_Inh_)(O^2−^,Vo)_4_, recognizing that point defects are always present in a growing oxide. The chemical formula in Figure 6a, (Fe^3+^, Fe^2+^)(Fe^3+^,Cr^3+^, Fe^2+^,Vm)_2_(Fe*_i_*^2+^,V_Inh_)(O^2−^,Vo)_4_, shows both presence of cation vacancies (Vm), cation interstitials (Fe*_i_*^2+^), and oxygen vacancies (Vo) for the growing Fe_3−x_Cr_x_O_4_ phase. The first and second sublattices denote the tetrahedral and octahedral interstices, respectively, and the fourth the oxygen sublattice. The third item stands for extra octahedral interstices which are inherently vacant (V_Inh_) for the stoichiometric composition. In the sublattices of *n* oxygen ions, the half of the *n* octahedral coordination was filled with cations. The other half of the *n* octahedral sites are called the extra octahedral interstices (inherently vacant), which provide space for the presence of cation interstitials, and would be occupied partly by cation interstitials when they are the predominant cation defects.

The defect distributions within the oxides are commonly dependent on the temperature, environmental oxygen partial pressure ($p_{o_{2}}$), and chromium content, as well as reactions occurring at the metal/oxide and oxide/environment interfaces [37]. Metal cation vacancies exist on octahedral sites at high $p_{o_{2}}$ while at low *p*_O2_ cation interstitials prevail. The critical boundary oxygen partial pressure ($p_{c,o_{2}}$) generally increases with temperature. Taking magnetite as an example, which has been studied extensively as a function of oxygen partial pressure, the critical boundary oxygen partial pressure ($p_{c,o_{2}}$) is ~10^−6^ atm at 1200 °C [48], ~10^−8.3^ atm at 1000 °C [46], ~10^−20^ atm at 600 °C [11], ~10^−25^–10^−22^ atm at 500 °C [11,46], and ~10^−30^ atm at 400 °C [11]. Previous studies have suggested that the rate-controlling step in the corrosion of ferritic-martensitic steels in SCW is the outward diffusion of iron and that the inner layer generally plays the role of the protective barrier layer [7,8,10,12,30,33,51]. However, the inner layer grows into the metal phase at the metal/inner layer interface [52,53], and the cations diffusing outwards through the inner layer are not involved in the growth of the inner layer into the metal. Therefore, the so-called rate-limiting step, to be more precise, actually refers to the outward transport of cations through the inner layer which is the slowest among all transport processes occurred within the scale. In the inner layer, cation interstitials are generally predominant at lower oxygen partial pressure within the deeper oxide layer, such as the inner layer [11,54].

Thus, from the view of improving oxidation resistance, i.e., by decreasing the diffusion flux of metal interstitials outward, more attention should be paid to decreasing the cation interstitials density, the kinetics of their generation at the metal/inner layer interface, and their diffusivity. The interstitial transport of metal cation in its own oxide generally follows an indirect interstitial diffusion mechanism. An interstitial cation pushes a lattice cation into the interstitial site. The net effect is the migration of one cation from one interstitial site to a different one, as shown in Figure 6b. During this process sufficient energy is required to break the bonds of the pushed lattice cation with all neighbors and to cause the necessary lattice distortions during the motion from one site to another. The higher is the required energy then the more difficult is interstitial transport. For the oxidation process, chromium contained in the F-M steels with a Cr content of 9–12 wt.%, generally is restricted to the inner layer and the oxides in the diffusion layer, and goes into the magnetite lattice octahedral sites replacing some Fe^3+^ and/or Fe^2+^, forming Fe_3−x_Cr_x_O_4_ spinel [55]. The octahedral sites occupied by Cr^3+^ are almost impossibly involved in the interstitials movement, because, relative to iron cations, chromium ions are possibly too tightly bound to be able to break their bonds with neighbors in the spinel structure [10], that is to say, more energy is required. The presence of chromium in the spinel structure decreases the number of available interstitial diffusion paths, as shown in Figure 6 from (b) to (c), thus inhibiting the outward transport of the cations interstitials, thereby slowing down the metal oxidation via the growth of the outer layer [48]. Additionally, the accumulation of cation interstitials at the metal/scale interface due to their decreasing outward transport, in turn, can restrain their generation reaction. However, upon the basis of 9 wt.%Cr in the substrate, a continuous increase in chromium content up to 12 wt.% may only result in a slightly limited-decrease of interstitial diffusivity within the inner layer of scales formed on F-M steels at temperatures lower than 600 °C, bringing about only a slight improvement in the oxidation resistance. Nevertheless, at 700 °C, the drastic enhancement in oxidation resistance of 12Cr steels relative to that of 9Cr steels can be attributed to the formation of a continuously protective chromia layer along the metal/scale interface [1,10,23]. For 9Cr steels and 12Cr steels, Nakagawa et al. [56] reported that at 650 °C a layer rich in chromium being 35 wt.% was present at the interface between the base metal and inner layer of scales on the 12Cr steel having markedly higher corrosion resistance but not for the 9Cr steel.

### 3.4. Oxygen Content and Flow Rate

**Figure 7:**
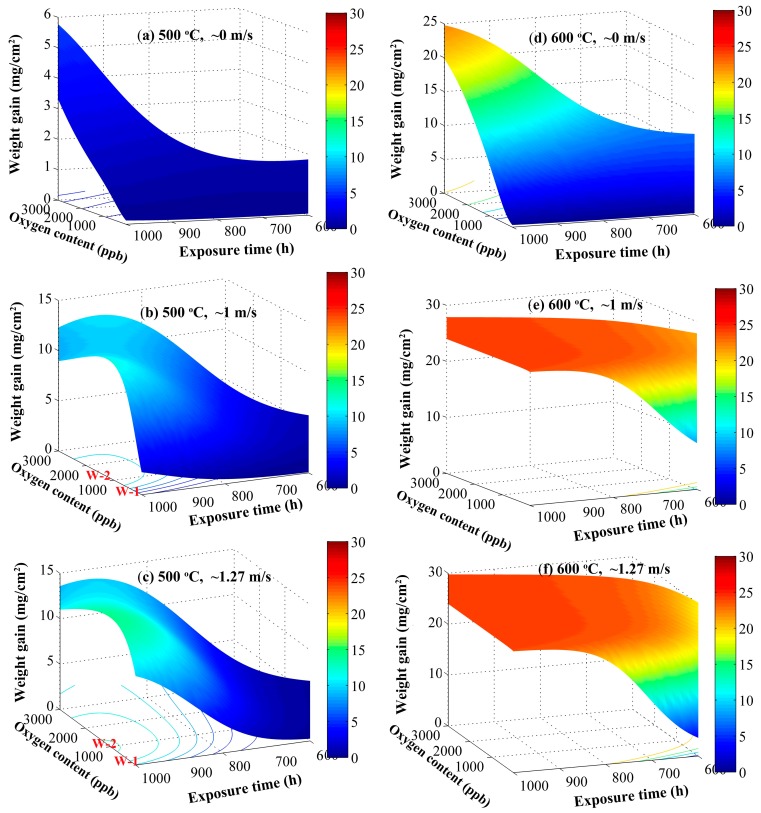
ANN-predicted values of weight gains vs. the oxygen content and exposure time at 500 °C and 600 °C under three flow rates. (**a**) 500 °C, ~0 m s^−1^; (**b**) 500 °C, ~1 m s^−1^; (**c**) 500 °C, ~1.27 m s^−1^; (**d**) 600 °C, ~0 m s^−1^; (**e**) 600 °C, ~1 m s^−1^; (**f**) 600 °C, ~1.27 m s^−1^.

Under three flow rates of ~0 m s^−1^, ~1 m s^−1^, and ~1.27 m s^−1^ at 500 °C and 600 °C, the predicted weight gains by the obtained ANN model are plotted as functions of the oxygen content and the exposure time, as shown in Figure 7. Figure 7a,d indicates that in the static systems (flow rate of ~0 m s^−1^), independent of the temperature of 500 °C or 600 °C, the weight gain increases with increasing oxygen content from 10 ppb to 3 ppm; a finding that is consistent with results obtained by Zhang et al. [13] in a static autoclave. Zhang et al. [13] showed that the weight gain of the F/M steel P92 increases with increasing dissolved oxygen for concentrations of 100–2000 ppb at 550 °C and at 25 MPa. Without hydro-mechanical disturbance in the static systems, the oxygen potential at the scale/SCW interface is generally lower than that in the bulk fluid due to the interfacial buildup of hydrogen released by the metal oxidation process [8,57]. The increase of oxygen content in the bulk of the fluid is beneficial in elevating the oxygen potential at the scale surface, thereby promoting the oxidation process, at least that induced by molecular oxygen (water also acts as an oxidant and in so doing results in the release of hydrogen at the inner layer/outer layer interface). However, the effect of oxygen content at 600 °C is more apparent than at 500 °C. When the flow rate is increased to approximate 1 m s^−1^ at 500 °C, there is a maximum weight gain obtained at an oxygen content of 600–2400 ppb for 1000 h (Zone W-2, see Figure 7b). The slight reduction of weight gain with a continuous increase in oxygen content from ~1.5 ppm to 3 ppm may be due to the formation of an additional protective hematite layer at the outmost surface and/or accumulated pores within the scale, which might prohibit cation diffusion. Zhang et al. [45] also reported that the weight gain for P92 was clearly higher at 2000 ppb DO than at 10 ppb DO in the flowing experiments. However, it is possible to speculate that the oxidation resistance of metals will inevitably decrease with increasing oxygen content, possibly at DO > 20 ppm [58], which is beyond the scope of present work and model, so that the corresponding, predicted results are not included in Figure 7. With a continuous increase of the flow rate from ~1m s^−1^ to about 1.27 m s^−1^, Figure 7c reveals the occurrence of the Zone W-2 at lower oxygen content compared with Figure 7b, implying that high flow rate may promote the formation of protective hematite and/or beneficial caves (hindering the lattice transport of cations and anions) at the lower oxygen content due to enhancing hydro-mechanical disturbance. Enhanced hydro-mechanical disturbances can promote to take the generated hydrogen away from the scale surface, elevating the oxygen potential at the scale/environment interface. However, there is a different phenomenon when the temperature is raised to 600 °C, as seen in Figure 7e–f. In this case, the oxygen content plays an almost negligible role. Thus, to a great degree, the effective oxygen potential at the scale/SCW interface may be determined together by the oxygen concentration, the flow rate of the SCW, and temperature, as predicted by the Mixed Potential Model [59].

**Figure 8:**
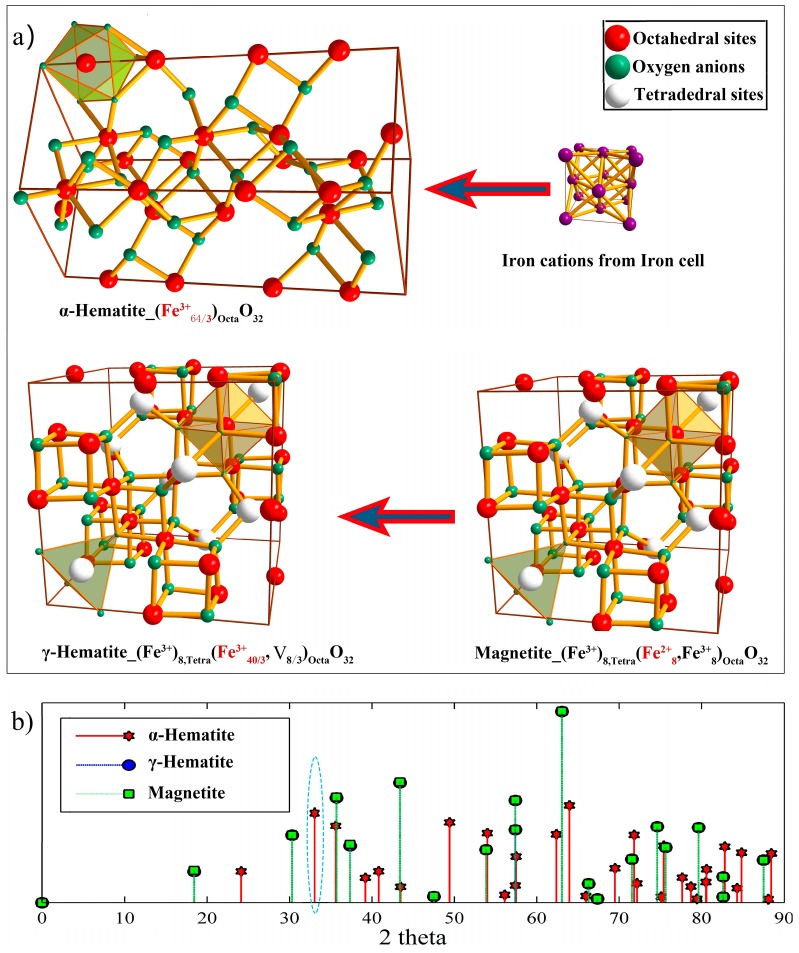
(**a**) Unit cell structure and possible generation ways of the predominant α-hematite and the γ-hematite, and (**b**) the theoretically-calculated XRD patters for the two hematite and magnetite. Note that XRD patterns are theoretically calculated according to the software of Diamond 3 with Cu-Ka1 being a type of X-ray.

In SCW and in high-temperature steam (below the critical pressure temperature of 22.12 MPa), it has been widely accepted that the concurrent growth mechanism of the formed oxide scales on iron-based steels generally occur both at the scale surface via cation diffusion outward and at the metal/scale interface [53,60,61]. The former translates that the outer magnetite layer very predominantly grows at the outer layer/environment interface, which was accompanied by the generation of the cation vacancies on the outer layer surface. The initial increase in the oxygen potential at the surface $\left(p_{s,o_{2}}\right)$ caused by an increase in the DO content and/or in the flow rate, generally accelerates the oxidation process, because it definitely promotes the generation of the cation vacancies on the outer layer surface. However, when the $p_{s,o_{2}}$ increases to an intermediate level, equivalent to the DO content of tens-to-hundreds ppb to several ppm depending on the temperature and flow rate, the minimum oxidation rate was observed. There are two possible processes that could be responsible for this phenomenon. On one hand, the occurrence of an additional, fourth, continous compact protective hematite layer at the outmost surface [3,4,62]. On the other hand, the existence of a certain amount of pores within the oxide scales strongly hampers the lattice diffusion of Fe cations outward [13,63]. In fact, the latter generally are also beneficial to the formation of hematite by second oxidation of magnetite [28,63]. In this case, the generated hematite should be cubic structure γ-hematite [${\left(Fe^{3+}\right)}_{8,Tetra}{\left(Fe_{40/3}^{3+},V_{8/3}\right)}_{Octa}O_{32}$] [45], as shown in Figure 8. However, our previous work and some available literature indicated that the characteristic peak located at 2θ ≈ 33°, belonging to α-hematite as depicted in the blue circled area of Figure 8b, was generally observed in X-ray diffraction (XRD) patterns of iron-based steels exposed to SCW with a dissolved oxygen content of several ppm [3,64], or in ppb level DO SCW for thousands of hours [65]. This phenomenon implys that the generated hematite may be predominant rhombohedral, corundum structure α-hematite with all the Fe^3+^ located on the octahedral sites [62]. This may be due to the conversation from γ-hematite to α-hematite because of lower stability of the former at higher temperatures [66]. With the continuous increase in the oxygen potential at the scale surface, samples will gradually develop more porous oxide scales to significantly promote oxygen/water transport inwards, especially with the occurrence of almost through-scale, connected pore networks in the entire scale [67]. Moreover, a high DO content more than thousands ppm easily leads to a decrease in the protective ability of the Cr-rich inner layer (Cr(III) in Cr-rich oxides being oxidized to soluble Cr^6+^ compounds) [67,68], cracks and exfoliation of the oxide scale [14]. Eventually, the protective effect of the oxide scales essentially also disappears completely.

## 4. Conclusions

This paper systematically performs the data mining and uncovers the non-linear relationships between the weight gains of 9-12Cr F-M steels in supercritical water and seven primary independent variables using a supervised artificial neural network (ANN). The principal findings may be summarized as follows:

- Fuzzy curve analysis ranks temperature and exposure time as the most important. However, the importance of the oxygen concentration comes in the last place. The obtained three-layer ANN architecture of 7:30:1 exhibits a satisfying performance with an R-squared value of more than 0.98.
- Predicted weight gain increases with temperature and exposure time, being in good consistence with experimentally measured data. The departure of oxidation kinetics from the parabolic law may be due to the dependence of the effective diffusivities of cations and anions through the oxide scale on short-circuit transport paths, such as the oxide grain boundaries. The dependence decreases with the temperature from 500 °C to 600 °C, because of the reduction of density of the short-circuit transport paths, due to the enlargement of oxide grain size. However, this dependence enhances with an increase in temperature up to 700 °C, deriving from the local formation of chromia within the scales at the high temperature. Additionally, high temperature delays the occurrence of the steady-state oxidation stage of 9-12Cr F-M steels in SCW.
- Influence of the chromium content (~9–12 wt.%) in the substrate on the weight gain is negligible at 500 °C, but becomes marked at 600 °C and above, due to the additional local formation of chromia. The certain amount of Cr restricted in the inner layer, can “block” some transport paths of cation defects (predominant cation interstitials) and thus inhibits the generation kinetics of cation defects, to decrease the diffusion flux of cations outward, finally improving the oxidation resistance of steels.
- At 500 °C, the oxygen content at which there is a maximum weight gain decreases with increasing SCW flow rate. The effective oxygen potential at the scale/SCW interface, being synergistically determined by oxygen concentration, flow rate of SCW and temperature, plays a key role in influencing the oxidation rate of steels. Higher oxygen potentials at the scale/SCW interface can promote the secondary oxidation of magnetite, accelerating the occurrence of hematite on the outmost surface.

## Figures and Tables

**Table 1:** Input parameters employed in the neural network systems.

Input variables	Range
temperature, °C	500–700
Chromium content, wt.%	8.37–12.12
ODS treatment	exists(1) or not(0)
surface treatment	exists(1) or not(0)
oxygen concentration, ppb	10–8000
exposure time, h	1–1000
flow rate, m·s^−1^	0–1.27
